# SARConnect: A Tool to Interrogate the Connectivity Between Proteins, Chemical Structures and Activity Data

**DOI:** 10.1002/minf.201200030

**Published:** 2012-08-07

**Authors:** Mats Eriksson, Ingemar Nilsson, Thierry Kogej, Christopher Southan, Martin Johansson, Christian Tyrchan, Sorel Muresan, Niklas Blomberg, Marcus Bjäreland

**Affiliations:** a Discovery Sciences, Computational Sciences, AstraZeneca R&D Mölndal, S-431 83 Mölndal, Sweden; b CVGI AstraZeneca R&D Mölndal, S-431 83 Mölndal, Sweden; c ChrisDS Consulting: http://www.cdsouthan.info/Consult/CDS_cons.htm; d AstraZeneca R&D Information, S-431 83 Mölndal, Sweden; e R&I AstraZeneca R&D Mölndal, S-431 83 Mölndal, Sweden

**Keywords:** Proteins, Activity data, Structure-activity relationships (SAR), Chemical structures

## Abstract

The access and use of large-scale structure-activity relationships (SAR) is increasing as the range of targets and availability of bioactive compound-to-protein mappings expands. However, effective exploitation requires merging and normalisation of activity data, mappings to target classifications as well as visual display of chemical structure relationships. This work describes the development of the application “SARConnect” to address these issues. We discuss options for delivery and analysis of large-scale SAR data together with a set of use-cases to illustrate the design choices and utility. The main activity sources of ChEMBL,^1^ GOSTAR^2^ and AstraZeneca’s internal system IBIS, had already been integrated in Chemistry Connect.^3^ For target relationships we selected human UniProtKB/Swiss-Prot^4^ as our primary source of a heuristic target classification. Similarly, to explore chemical relationships we combined several methods for framework and scaffold analysis into a unified, hierarchical classification where ease of navigation was the primary goal. An application was built on TIBCO Spotfire to retrieve data for visual display. Consequently, users can explore relationships between target, activity and structure across internal, external and commercial sources that encompass approximately 3 million compounds, 2000 human proteins and 10 million activity values. Examples showing the utility of the application are given.

## 1 Introduction

In medicinal chemistry, the analysis of structure-activity relationships (SAR) is of fundamental importance in understanding the structural determinants of biological activity, and it underpins lead generation for drug development. Although the dominance of mono-target approaches has been challenged by polypharmacology and regulatory networks, a thorough understanding of molecular SAR and selectivity remains a key driver for most medicinal chemistry projects.^5^ In drug discovery the use of molecular target classifications, such as GPCRs, kinases, proteases, NHRs and ion channels, became widely adopted as the human genome was approaching completion.^6^ Postgenomically, these were given a formal descriptive framework in the landmark “Druggable Genome” paper.^7^

During the past decade the medicinal chemistry community has witnessed a rapid growth (via their own collective output) in public SAR data from patents, journals and repositories such as PubChem BioAssay and ChEMBL.^8^ This has extended the range of proteins being explored as targets for possible therapeutic modulation and also includes cross-reactivity data generated from panel screening. However, none of these would claim complete capture and most institutions have a repository for proprietary internal assay results.

The consequent necessity to mine across multiple sources is demanding for both bench scientists and informaticians because optimally exploiting any individual database needs a significant time investment, not only to understand the data structure, query options and content but also to develop post-processing filtering strategies. These problems are compounded when data needs to be extracted and merged from different sources. Assembling and maintaining these resources poses significant technical and organisational challenges. In particular, the need to query across resources has highlighted the challenges of interoperability and the need to collaborate across industry, academia and learned societies to establish long-term solutions.^[9]^ Navigating the resulting extended SAR matrix presents new challenges in terms of both volume and complexity of data. Several applications have been described for summarising SAR data, either as tables^10^ or networks.^11^ They include browsing and filtering hierarchies of compounds (e.g. built on molecular topology or structure similarities) and targets (e.g. target ontologies of different levels).^12^

Within AstraZeneca (AZ) we conceived an application to meet these challenges. This was predicated on the success of Chemistry Connect that already provided the first level of comprehensive data integration across multiple sources.^3^ We specified that this should be able to: i) retrieve integrated SAR data, ii) connect this to individual proteins and their target classes, iii) use gene/protein identifiers to connect to the biology around targets, iv) connect targets via chemical scaffolds-in-common, and v) provide navigable hierarchies for both target classes and scaffolds. In this paper we present the development of SARConnect, a client application built on TIBCO Spotfire that efficiently retrieves data for visual display and allows users to navigate these relationships. We also developed a heuristic target classification to support the browsing and retrieval of related targets for medicinal chemistry users. This is a critical component of navigation and query systems for molecular databases and while, as outlined below, there are no ‘correct’ solutions we hope that the availability of analogous classification systems in public domain resources such as ConceptWiki will support interoperability between both public and proprietary data sources.^13^

## 2 Methods

Our implementation of the target and target-class mappings was based on a number of simplifying assumptions, thus:

– A default assumption for internal usage that gene=protein.
– Include a complete set of canonical human proteins as the top layer.
– Use Swiss-Prot as a single source to extract a middle layer of target-class mappings between protein IDs and compounds in our data sources.
– Split complex targets into their constituent protein IDs.
– Ensure we could utilise the powerful query options orthogonal to target classifications such as Enzyme Commission numbers, the Human Gene Nomenclature Committee (HGNC) subfamily symbol stems,^14^ the Gene Ontology^15^ functional categories and the InterPro^16^ homology-based classification of families and domains.

Human proteins were collected as 1:1:1 entries with HGNC, Entrez Gene^17^ and Uniprot/Swiss-Prot IDs. To provide an overview of the accessible target landscape, we focused on three major classes: enzymes, G-protein coupled receptors (GPCR), ion channels and the fourth and smallest class of nuclear hormone receptors (NHR). Human proteins not belonging to these are classified as “Other”. An initial analysis revealed that a hierarchy with three levels was sufficient to cover all relevant target information and facilitate comprehensive activity data mapping. The content was further annotated with keywords such as kinase, lipase or transmembrane etc. Because maximising the chemistry mapping was the objective, we emphasised recall for the protein classifiers rather than being concerned about equivocal or multiple memberships. We thus applied “greedy”, high capture, selections, such as transporters that would also include channels. We also selected all EC numbers and PDB structures. As expected, many entries have multiple memberships (e.g. protease, serine protease, EC number and PDB). We used one exclusion list for class 1 GPCRs by intersecting “G-protein coupled receptor” family, with “olfactory” from the Web resource cross-reference to the Human Olfactory Receptor Data Exploratorium, (HORDE).^18^ After evaluations via the UniProt web interface the family information from an internal XML instance of human Swiss-Prot was extracted into a local Oracle database of 19 426 records with a Pipeline Pilot interface. We have deposited the structural classification as an Excel file and technical details of the target database as Supplementary Data. A summary of the occupancy figures in the database are found in Results. In brief, we provided three principal levels for users to navigate. The top level consists of broad target classes, encompassing approximately 4600 proteins in four major classes, with 14 800 human proteins classified as “other”, thus adding up to 19 400 in the protein classification DB. The second level consists mainly of the Swiss-Prot family designations and the third level is sub-families along with EC number sub-groups (for further details on technical description see Supporting Information).

### 2.1 Chemical Structure Classification

**Figure 1:**
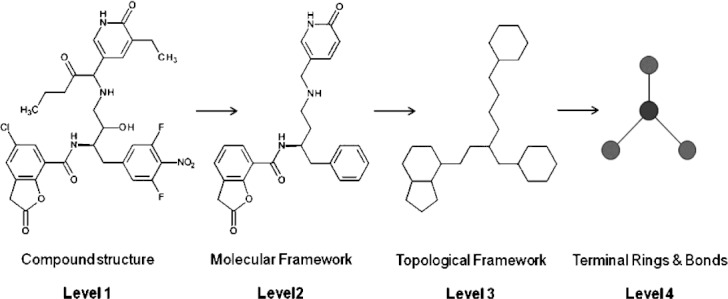
Four distinct levels of hierarchy for structure classification.

The structural classification is represented as a four level hierarchy (Figure 1) similar to the approach described by Bemis and Murcko.^19^ The first level corresponds to the compound structures standardised according to AZ in-house chemistry business rules.^3^ For the second level, molecular frameworks are generated by removing terminal groups and side chains. In the third step, topological frameworks are prepared by removing exocyclic double bonds and double bonds directly attached to the linker and ignoring all bond types and atom types. The fourth level (Top Level) classifies a set of molecules into limited number of sets defined by terminal rings and bonds. This latter classification schema has proved successful in differentiating between drugs, clinical candidates and bioactive molecules.^20^

### 2.2 Test and Results

The integration of SAR data for AZ in-house applications has been described recently.^3^ The current application incorporates the web-service interface from Chemistry Connect. The Test and Results was specifically focused on integrating the three major SAR sources: IBIS (internal), GOSTAR (commercial) and ChEMBL (public).

Activity values for all three sources are transformed into a normalized potency (pAct) taken as the negative logarithm of the potency converted to molar concentration. The “% effect” results are transformed to pAct by assuming a concentration-response curve from 0 at the bottom and 100 at the top with a slope of 1. While this approximation has known caveats, SARConnect users always have direct links to the untransformed data and the primary documents or assay records via Chemistry Connect. Thus for GOSTAR and ChEMBL all results with endpoints of EC50, IC50, Ki, potency or % inhibition standard activity types are captured whereas binned or cut-off values are not. A similar parsing scheme is applied to in-house data (IBIS) where the extraction is limited to the subset of test records internally flagged as active. Test records are marked as active if the converted pAct is greater than or equal to 5.0.

### 2.3 Technical Description SARConnect Application

SARConnect is built using the TIBCO Spotfire 3.1 platform.^21^ All functionalities are included in a TIBCO Spotfire analysis document (.dxp), which can be loaded in the TIBCO Spotfire client or in a browser with the TIBCO Spotfire Web Player. The application is designed to allow users to query SAR data from several different entry points and explore large data sets. This is achieved by using built-in TIBCO Spotfire functionality extended by the IronPython scripting interface.^22^ These extensions allow data extracted from Chemistry Connect web-services to be merged with other sources.

## 3 Results and Discussion

### 3.1 A Revised AZ-Wide Target Classification

Historically, AZ had utilised a number of target class listings in different parts of the organisation. This presented problems where data-analysis was no longer confined to a specialised computational function but became part of the wider medicinal chemistry and bioscience practice. The lack of internal consistency between systems and the ad hoc usage of different gene and protein names gave rise to continual cross-mapping ambiguities (i.e. “which target did you mean”) which hampered effective analysis of the target landscape. This affected many areas including target portfolio management, project titles, disease-to-gene associations, designations for prospective HTS runs and assigning names to the thousands of different in vitro target assays used by project teams. In addition insufficient internal maintenance inevitably caused these classifications to decay. This resulted in target number differences as external sources updated and cases of the internal usage of symbols and names long after they had been superseded by HGNC approved revisions. This is exacerbated by the sustainability of some public target databases because even if they stayed “live” funding fluctuations can constrain update frequencies.

The classification of drug targets is related to the wider challenge of dividing up proteomes into structural and functional groupings that have utility for classification and make biological sense. A protein ontology with this objective has been developed but was not primarily designed for predictive medicinal chemistry.^23^ An inspection of the top-500 protein family automated annotations for the human genome illustrates the target-specific challenge.^24^ This includes 759 rhodopsin-like GPCRs, 481 Serine-threonine/tyrosine-protein kinases, and 121 chymotrypsin-type S1A proteases. These families cannot be rooted in the homology sense. However, kinases and the serine proteases can be sequence-clustered with subfamilies at the leaves of each tree. For the GPCRs the term clan is used for group of families. While there are indications of evolutionary relationship (via genomic duplications from a common ancestor) the sequence similarity across the clan is insufficient to root the clusters.^25^ To make an analogous classification with proteases is even more complex because, despite being unified under Enzyme Commission 3.4. as hydrolases acting on peptide bonds, they have many different evolutionary origins.^26^ The GPCR families also illustrate the problem of progressive classification shifts during continued recuration. For example, one of the earliest post-genomic analyses had grouped 342 non-olfactory human GPCR sequences into five main families: glutamate, rhodopsin, adhesion, frizzled, and secretin, with the rhodopsins further subdivided into four groups and 13 sub-families.^27^ Subsequent reviews are largely congruent with this classification but inevitably present differences.^28^

Orthogonal classification systems are found in dedicated specialist databases: these include GPCRDB,^29^ a molecular-class information system that collates and validates heterogeneous data, the GPCR section of the International Union of Basic and Clinical Pharmacology (IUPHAR) organisation, the GPCR spatial restraint resource for structural modelling and the GPCR-Oligomer Knowledge Base.^30^ Yet another level of connectivity is interposed via links from these family databases to the major pipelines that encompass all proteins, such as Ensembl,^31^ Entrez Gene, HNGC and UniProtKB. Efforts are underway to enhance connectivity still further by integrating GPCRDB with new methods for exploring, visualising and live-linking journal articles via the Utopia PDF reader.^32^

As annotation and cross-referencing continues on a global scale it can result in inter-source discordances, family size changes, asynchronous updates, differences in curation rules, redundancy and circular connectivity that obscures data provenance. However, these challenges reflect the reality of a progressive evolution of protein classification and the collated analysis of a large expert community. While GPCRs have historically received a lot of attention other target classes are similarly endowed with specialist resources. A sample would include the NucleaRDB for nuclear receptors,^33^ the MEROPS database for proteases,^34^ substrates and inhibitors, annotation of human and mouse kinomes in Swiss-Prot,^35^ the Transporter Classification Database (TCB), the IUPHAR^36^ Guide to Receptors and Channels (GRAC) and a recent review of histone deacetylases (HDACs).^37^

Our solutions in the context of developing SARConnect were guided by pragmatic principles. The first was to have real-world utility that chemists, biologists and portfolio managers should find easy to use. This led us to develop a “flat” hierarchy with a small number of classes and subclasses that do not necessarily reflect a detailed evolutionary classification but can be easily navigated by non-experts. The second was to reduce maintenance overheads that a complex system abstracted and integrated from many sources would necessitate. The third was to use simplifying assumptions but understand their caveats and document their consequences. From an internal assessment we noticed recent improvements in sequence features, cross-references, keywords and other annotations in human Swiss-Prot, largely due to the Human Proteomics Initiative (HPI) and its successor the Chordata protein annotation program.^38^ A brief summary of the target classes in our three-level hierarchy is given in Table 1.

**Table 1:** Target statistics for classification in SARConnect.

Target class	Count
G-protein coupled receptor	827
G-protein coupled receptor (Class A)	717
G-protein coupled receptor (Class B)	49
G-protein coupled receptor (Class C)	22
Kinase	608
Nuclear hormone receptor	48
Ion-channel	227
Lipase	40
Phosphatase	180
Protease	575
.. Aspartyl	19
.. Cysteine	153
.. Serine	241
.. Metallo	187
.. Threonine	29
Transporter	538
EC number	4001
PDB entry	4436

### 3.2 Chemical Structure Classification

In the context of medicinal chemistry, structure classification is performed to rationalise SAR for a chemical series according to the concept that “similar structures have similar bioactivities”. Analysis of compound clusters and near-neighbours can be performed using a range of fingerprints derived from molecular connectivity tables and/or sets of physicochemical properties and similarity metrics.^39^ Such analyses are computationally intensive and the cluster space (i.e. the compound hierarchy) changes with additional compounds. In addition, typical clustering methods do not offer a high enough level of structure abstraction for efficient visual browsing of large sets of compounds.

For SARConnect we aimed to define a compound hierarchy that complemented our target classification and facilitated the answering of specific questions such as “retrieve all compounds that modulate target P” as well as more general queries such as “retrieve all chemical series for kinases”. Such hierarchies can be built using molecular scaffolds and refinements based on ring and linker concepts.19a, 20 For instance, Scaffold Hunter, a tree-like hierarchical representation for chemical space navigation,^40^, has been used to analyse natural products and “target hopping” approaches. The classification we have in SARConnect is conceptually similar to these systems with modifications implemented primarily to aid navigation and simplify compound grouping in the TIBCO Spotfire interface. Table 2 shows a summary of the statistics for the structural hierarchy in the three sources, IBIS, GOSTAR and ChEMBL.

**Table 2:** Compound and structural classification statistics for the sources of test data in SARConnect. The unique number of Topological and Molecular Framework are calculated with respect to the other two data sources in the table. The percentage is related to that proportion of the whole set of compounds for Topological Framework and Molecular Framework, respectively.

	IBIS	GOSTAR	ChEMBL
Compounds	1 180 457 (38 %)	1 547 979 (49 %)	399 836 (13 %)
Top level	9	9	9
Topological Frameworks	75 002 (31 %)	132 874 (55 %)	34 074 (14 %)
Molecular Frameworks	373 606 (38 %)	471 818 (48 %)	129 159 (13 %)
Unique top level	0	0	0
Unique Topological Frameworks	44 379 (30 %)	93 588 (65 %)	6705 (5 %)
Unique Molecular Frameworks	308 613 (41 %)	389 824 (52 %)	54 064 (8 %)

**Figure 2:**
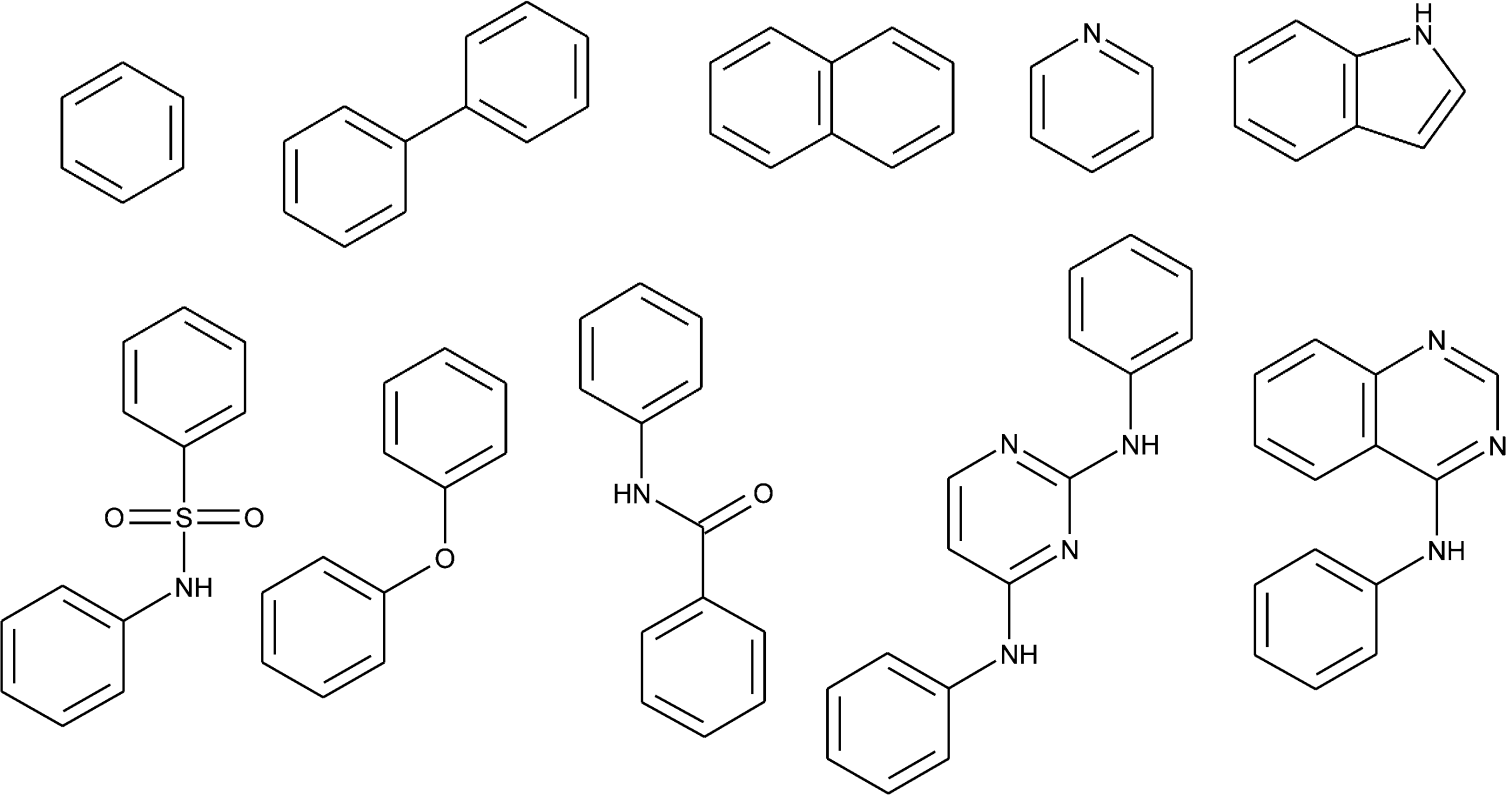
Top-ten most frequent molecular frameworks in SARConnect.

The Top Level classifier is used as an initial filter for further exploration using the Topological Framework and Molecular Framework. The number of compounds from GOSTAR (49 %) is larger than in IBIS (38 %) and ChEMBL (13 %) and this is also reflected in the number of Topological Frameworks and Molecular Frameworks. The percentage of Topological Framework and unique Topological Framework are higher in GOSTAR relative to IBIS, which reflects the wider set of targets covered in this source. Interestingly, the number of Molecular Framework and unique Molecular Framework is comparatively larger in IBIS than in GOSTAR. This probably reflects small variations of a scaffold (e.g. a phenyl ring is replaced by a pyrimidine ring) that are not necessarily published in a patent. Analysis of the top ten most frequent Molecular Framework with test data in SARConnect are shown in Figure 2.

Along with expected common smaller scaffolds, larger ones can be found that we might predict to exhibit poly-pharmacology. For example, the large N2,N4-diphenylpyrimidine-2,4-diamine scaffold, is present in drugs such as Rilpivirine, targeting HIV reverse transcriptase, Pazopanib, a multikinase angiogenesis inhibitor, as well as in compounds in phase II trials such as TG-101348 inhibiting Janus kinase 2. As the second most frequent scaffold with activity data this scaffold is linked to 26 900 compounds.^41^ Of these, 14 700 compounds have a pAct ranging from 5.0 to 10.0 for 416 targets covering all classes.

### 3.3 SAR Data

As a step towards broadening and simplifying AZ’s exploitation of multiple sources we recently developed the enterprise Chemistry Connect application.^3^ This integrates 55 million unique chemical structures from 20 internal and external data sources. It also includes reported bioactivity assay data merged into a set of tables designated “Test and Results” (T&R). The first version of this included links from 4.5 million compounds, via 10 000 protein identifiers, to 12 million in vitro SAR data points and other types of bio-annotation such as in vivo pharmacological activity. As a prelude to the development of SAR-connect we implemented a second T&R version with increased activity stringencies and omitting some sources that we determined as having promiscuous target mappings. Also, specifically for SAR-connect, we introduced the additional restrict of human proteins to the normalization and integration rules for the three sources. The resulting aggregated and comparative statistical analysis of content for SAR-connect content is shown in Table 3.

**Table 3:** Target, compound and activity statistics for the three sources of test data in SARConnect. The unique number of targets and compounds are calculated with respect to the other two data sources in the table.

	IBIS	GOSTAR	ChEMBL
Targets	835	4785	2514
Human targets	835	2424	1298
Unique targets	63	2626	587
Activity records	4 186 903	4 235 360	1 255 670
Compounds	1 180 457	1 547 979	399 836
Unique compounds	1 083 677	1 367 476	247 923
pAct average, median, 90th percentile	5.0, 4.9, 6.4	6.1, 6.0, 8.2	5.3, 4.9, 7.2

While a detailed comparison is outside the scope of this report we note that activity records cannot be directly compared because they are not standardized. The substantial proportion of unique chemical structures in each source suggests complementary chemotype coverage. The somewhat lower novelty of ChEMBL is explained by extractions from a proportion of the same journals in GOSTAR. This is also the reason for lower target novelty in this set because GOSTAR includes both journals and patents. Given the initial disclosure of targets in the literature and/or patents the small proportion of novel targets becomes explicable. Some of these may also be for specificity testing. Given that targets are proportionally less unique than compounds, it would indicate different compounds being tested against targets-in-common thus aggregating chemotypes across targets.

#### 3.3.1 The SARConnect Application

The development of the SARConnect has been driven by the need to efficiently retrieve and present SAR across target and target classes. The Target classification (see Methodology) linked to the application provides the biological dimension. This is matched by the four level structural classifications and the third dimension is represented by reprocessed SAR data. The pAct descriptor enables comparisons over different biological assays and their endpoints. The given mode of action (e.g. inhibitor, agonist, antagonist) and the original assay endpoints (e.g. *EC*_50_, *IC*_50_, *K*_i_) provides the trace back to original data and in combination with the other descriptors retrieved this creates the scene for the SARConnect application.

### 3.4 Data Retrieval

SARConnect allows data to be extracted and explored using different web services in Chemistry Connect.

1) Via target identifiers

One or several targets can be selected as prime target from the target classification. Users can choose to extract only SAR data for the selected targets, or to include all off-target SAR data for compounds with test data linked to the target selection.

2) Via compound structural information

Two different methods for extracting SAR data from a compound query structure are provided. All available SAR data in for compounds with either a matching substructure or a Lingos-based^42^ similarity value within a given threshold to the query structure can be loaded for visualization.

3) Extracting SAR data from patent identifiers

Given a list of patent numbers, document metadata, as well as all available SAR data for the claimed compounds will be loaded for visualization.

### 3.5 Data Processing and Analysis

SARConnect handles large sets of hits efficiently. For example, a query for all reported kinase SAR will retrieve ∼1.5 M records into the TIBCO Spotfire interface but still provides interactive analysis. SARConnect also provides a set of pre-calculated physico-chemical properties such as molecular weight (*MW*), *c*Log*P*, polar surface area (*PSA*), number of rings, activity value, activity flag, mechanism of action, data source, compound classifications and target classifications. These can be used as data reduction filters.

### 3.6 SARConnect

**Figure 3:**
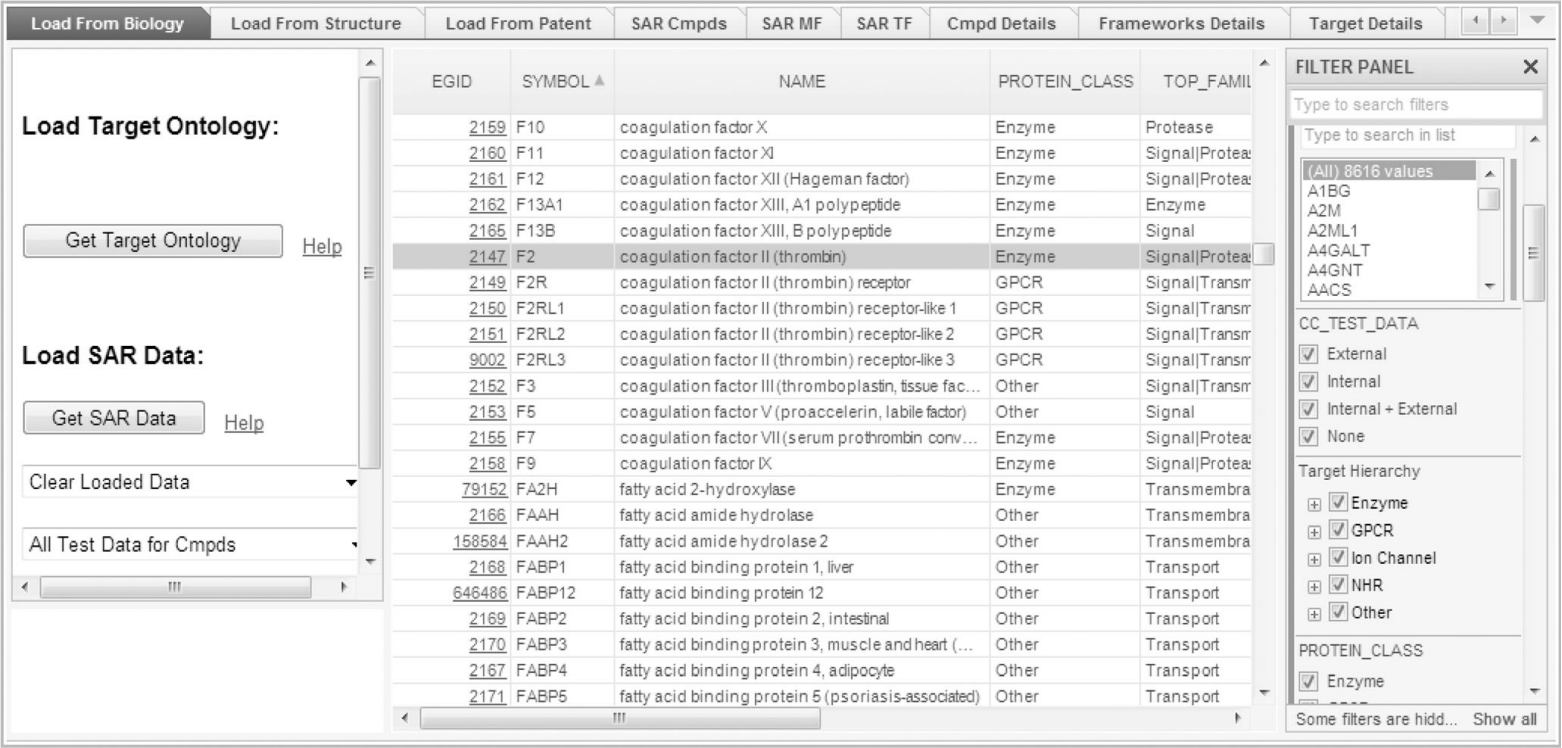
A view of the target (class) selection interface of SARConnect using TIBCO Spotfire.

SARConnect enables biology- and chemistry-centric searches with the three entry points presented, namely from proteins, chemical structure or patent information (Figure 3). The target classification has a link-out to Entrez Gene ID and includes the HGNC symbol and HGNC full name as well as the target class membership. One or more targets can be selected as the primary query to which all retrieved SAR data will relate. The relationship of target and result can be restricted to the selected targets or extended to all cross screening results linked to the initially selected compounds. This facilitates an immediate indication of potential polypharmacology, selectivity or safety issues.

## 4 Practical Use – Cases

### 4.1 Extracting SAR Data from Target Identifiers: Thrombin Molecular Pharmacology

As a first example, a search for compounds screened against thrombin (Approved symbol F2, Gene ID: 2147) is presented, a serine protease that has been pursued as an anti-coagulation target for more than 35 years.^43^

**Figure 4:**
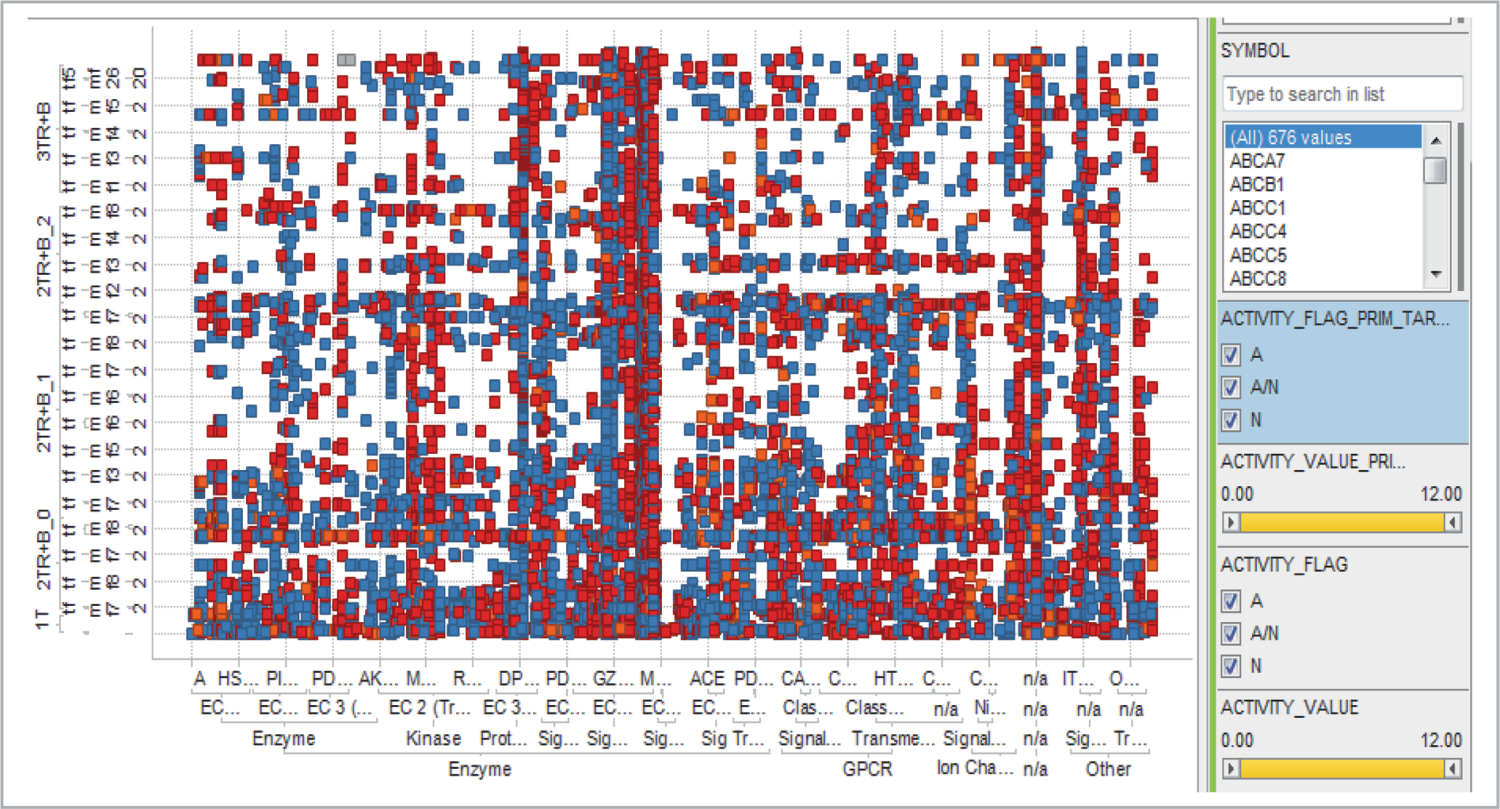
Extended SAR matrix of compounds linked to human thrombin. Compounds classified as active or inactive are coloured in red and blue, respectively.

The search resulted in ∼105 K records with 33 500 results linked to a thrombin assay result, including both positive (thrombin inhibitors) and negative results. The retrieval also captures 68 500 cross-screening records on the 676 additional targets of different classes. It can be assumed that much of this is selectivity screening of potential leads against other serine proteases (e.g. F10, F7, PLAU and ELA2). The SAR compounds view (Figure 4) displays the proteins against the chemical structural classification of the retrieved data. Removing records classified as non-active for the primary and cross-screening targets reduce the data to 58 K records covering 15 K compounds and 367 targets.

**Figure 5:**
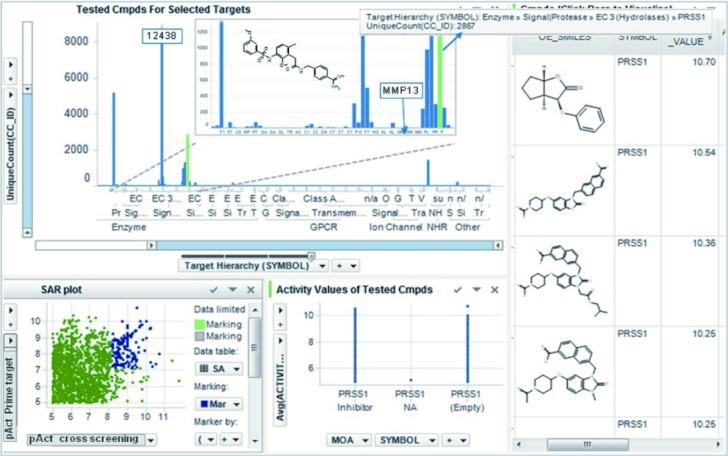
SARConnect target detail view.

This result reflects a general aspect of target query results that, before the introduction of SARConnect, were not possible for AZ scientists to visualize at this scale. It also highlights a major challenge for the design of selective compounds. We thus envisage SARConnect becoming an essential first step in AZ drug discovery projects because it quickly reveals pre-existing data relationships, including secondary pharmacology which would need to be addressed with off-target screens. Nevertheless, a full assessment of chemical safety risks requires additional data and specialised tools.^44^ In the next step, non-actives are removed and restriction on pAct >5, *MW*<600 and −2<*c*Log*P* <6 are applied, which reduces the thrombin data set to 48K records (see Figure 5).

The target view reveals the unique compound count for each target (upper left panel in Figure 5). Thrombin, as the prime target of our search query, is connected with all compounds (∼12 400) followed by Factor Xa, another serine protease acting prior to thrombin in the coagulation cascade. This enzyme is connected to 5200 unique compounds with recorded activity values. The expanded bar chart details the set of the overall thrombin active compounds modulated enzyme targets, many of them closely related serine proteases. The bar in green corresponds to trypsin (PRSS1), commonly included in serine protease selectivity screening. Further analysis also reveals the less obvious activity against matrix metallopeptidase 13 (MMP13) for a set of 25 compounds. The in-house designed thrombin inhibitor *N*-[(4-carbamimidoylphenyl)methyl]-2-[2-hydroxy-3-[(3-methoxyphenyl)sulfonylamino]-6-methyl-phenyl]acetamide, has a pAct of 7.71 and 5.79 on thrombin and MMP13, respectively.^45^

The option of visualising the molecular structure of compounds (as shown in the chemical structures of the blue records on the lower left scatter plot displayed in the right panel spreadsheet in Figure 5) enables the rapid inspection of the SAR for a primary target against any cross screening target.

### 4.2 The Framework Details Panel: Highlighting the Structural Diversity of Thrombin Inhibitors

**Figure 6:**
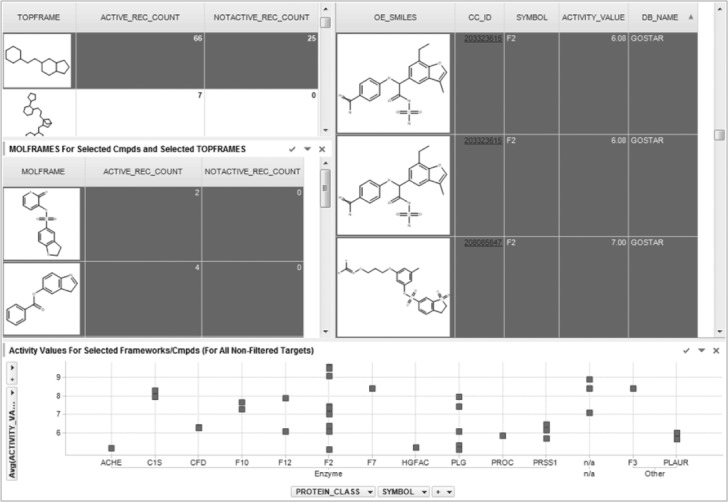
Molecular frameworks panel of selected thrombin actives. Topological frameworks are presented in the upper left panel. The mid left panel shows the molecular frameworks corresponding to the marked molecular topology and the upper right panel shows the corresponding compounds and associated pAct values. The lower panel shows the cross screening of the filtered compound set.

Key medicinal chemistry issues in the development of thrombin inhibitors include the molecular geometry and topology for anti-parallel beta-strand mimics as well as opportunities to extend interactions to the prime-side target pocket. These structural relationships can be analysed in a framework-centric view (see Figure 6).19a A total of 91 records, with 66 actives and 25 non-actives, delineate a distinct topology framework linked to thrombin. This relates to 23 compounds with 8 molecular frameworks and activity data for more than 10 distinct targets.

Compounds selected by their molecular frameworks can be further inspected with respect to their SAR data. Following the current example, the lower panel of Figure 6 shows for the selected compounds a pAct range from 5 to 9.3 on the prime target thrombin (F2) and low to high activity for plasminogen activator urokinase receptor (PLAUR), HGF activator (HGFAC), acetylcholinesterase (ACHE).

### 4.3 Extracting SAR Data from Compound Structural Information: Privileged Motifs

For the next use-case, we have queried SARConnect with spiro[indoline-3,4′-piperidine] scaffold, a known privileged motif for GPCR binding, to retrieve ∼6700 structures.^46^

**Figure 7:**
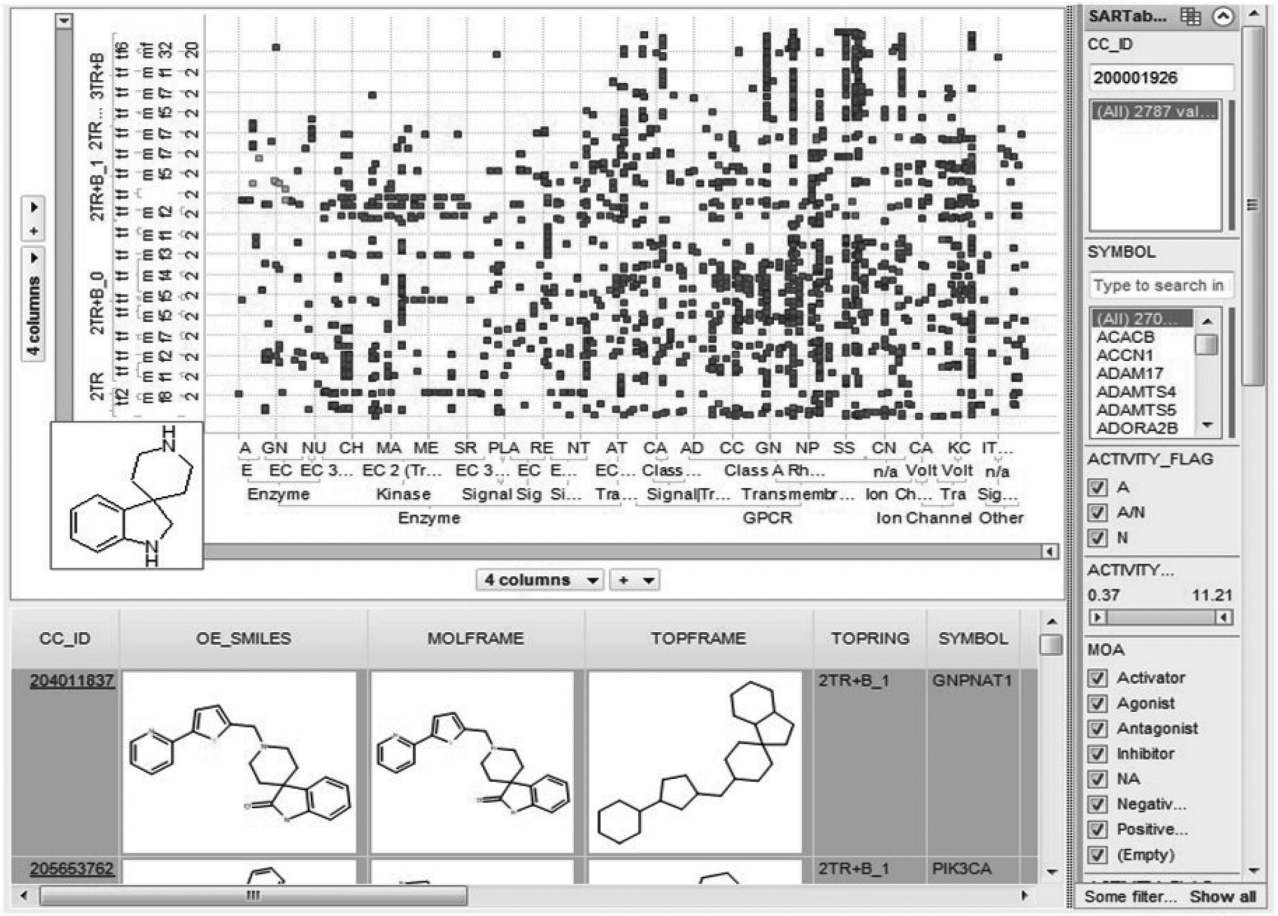
Results from a substructure search with spiro-indoline ‘privileged’ GPCR motif showing broad activity across target-classes, independent of topological context.

Extracting SAR data for these compounds gives ∼7000 records covering ∼2900 compounds (see Figure 7). Thus ∼4000 compounds do not have any biological data associated with them in SARConnect.

**Figure 8:**
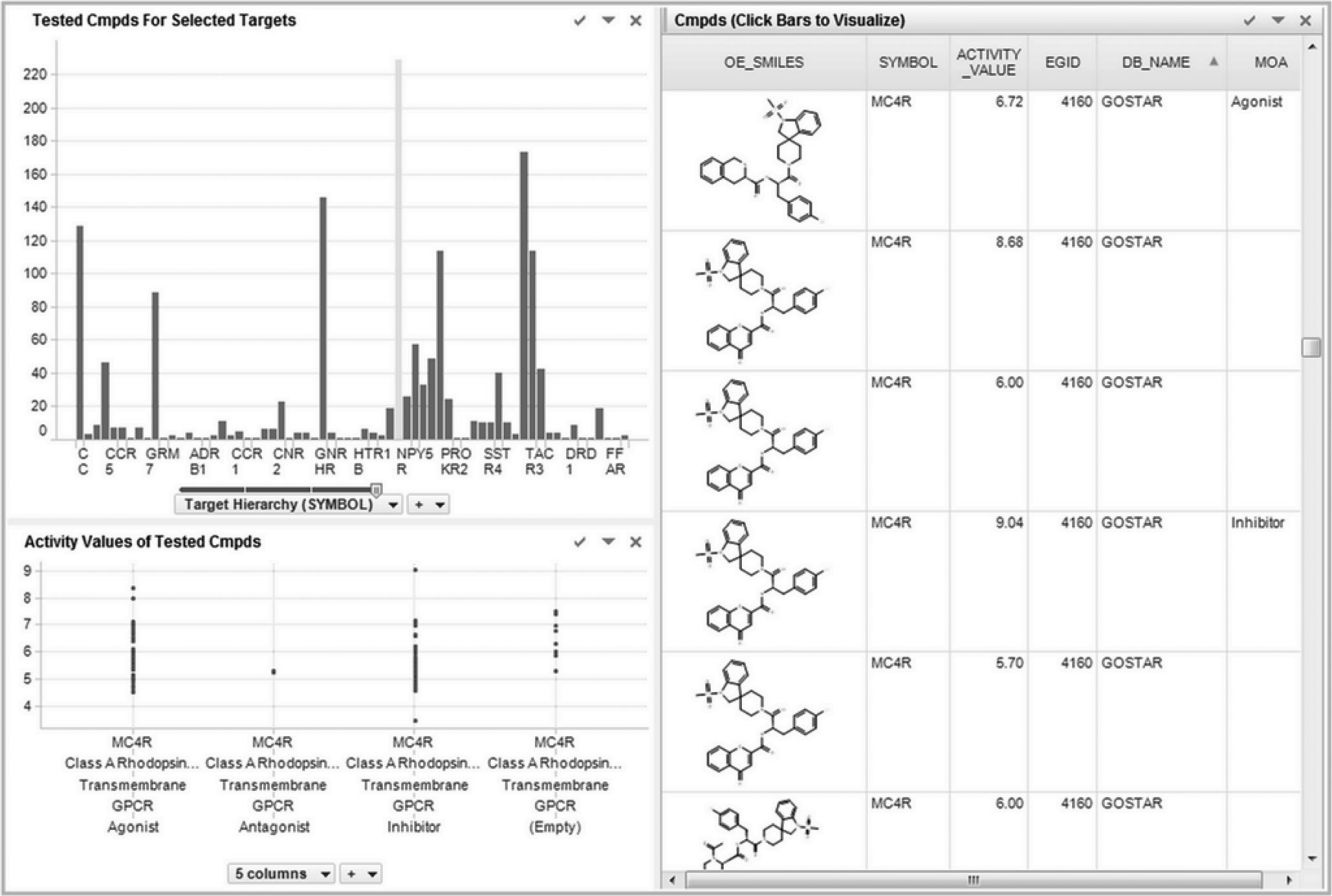
Target detail view for spiro[indoline-3,4′-piperidine] scaffold. The lower left panel shows the distribution of pAct values according to registered mode of action measured in the bioassay (MOA) and the right hand spreadsheet displays the records of the marked bar.

Sixteen GPCR targets have recorded activity on more than 20 compounds. MC4R has 657 records connected to 220 unique compounds- (see Figure 8). Five targets TACR1/2, OPLR1, GSHR1 and CCR2, all belonging to Class A, have data from more than 100 compounds. The only GPCR with more than 20 hits (89) which does not belong to Class A is the Class C glutamate receptor, metabotropic 2 (GRM2).

**Figure 9:**
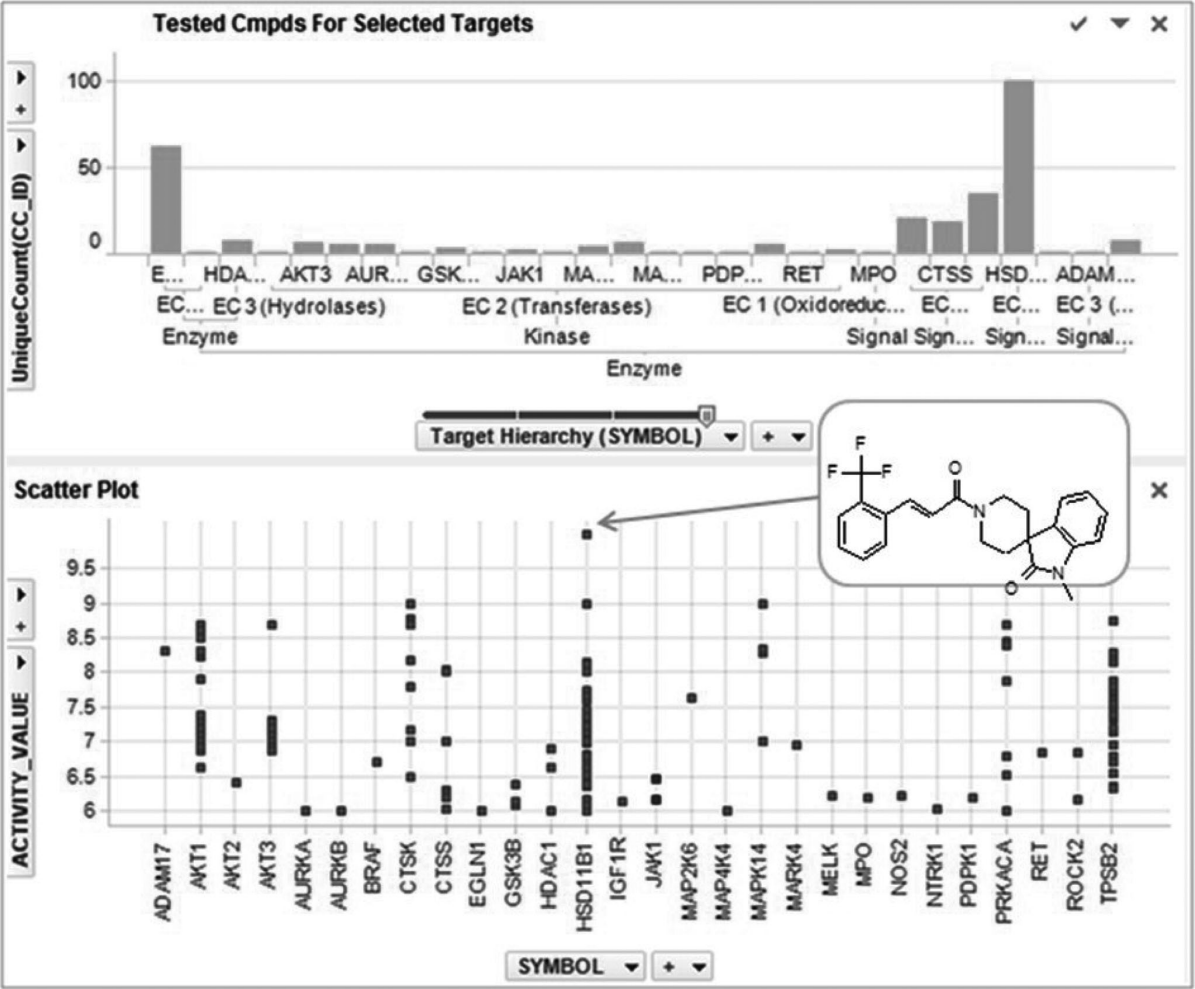
Detailed activity view for compounds with enzymatic activity having the spiro[indoline-3,4′-piperidine] scaffold.

Overall, the spiro[indoline-3,4′-piperidine] scaffold has been screened against all captured targets classes. Although a predilection for GPCRs is clear the application shows this structural motif is not selective. In total 2788 records are linked with SAR data towards the GPCR target class, for a total of 1219 unique compounds (Figure 8). Approximately ∼25 % (770) show activity against enzymes. Within the 770 compounds, 262 have a pAct value of above 6.0 on 28 enzyme targets, and greater than 9.0 on three targets (cathepsin K (CTSK), hydroxysteroid (11-beta) dehydrogenase (1HSD11B1) and mitogen-activated protein kinase 14 (MAPK14)). The compound 1-methyl-1′-[(*E*)-3-[2-(trifluoromethyl)phenyl]prop-2-enoyl]spiro[indoline-3,4′-piperidine]-2-one has a pAct of 10 on 1HSD11B1 (see Figure 9). Another 184 unique compounds have SAR data for ion channels, but only six have a pAct greater than 6.0. From this analysis the absence of SAR data for NHR receptors and spiro[indoline-3,4′-piperidine] motif is a potentially important observation.

**Figure 10:**
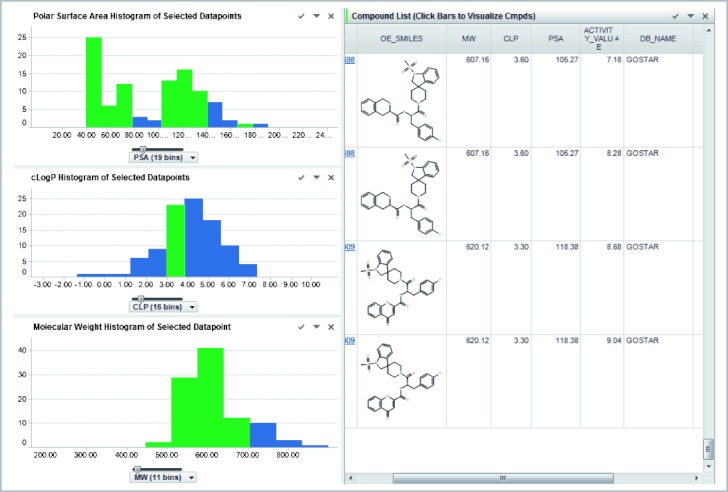
Physicochemical property distributions for sets of compounds selected in SARConnect.

Typically, MC4R agonists are large and lipophilic. Figure 10 shows this in the display of *c*Log*P*, PSA and MW for the set of compounds active against MC4R. In general, physicochemical properties are strongly correlated with DMPK, safety issues and attrition in clinical trials. Many studies have addressed these relationships and monitoring parameters such as Log*D* is a key requirement in compound design.^47^ Even for targets with a distinct preference for lipophilic compounds, it has been shown that one can find clinical candidates and drugs with physico-chemical properties in the usual drug-like corresponding ranges. SARConnect facilitates such investigations by displaying the properties of any selected set of compounds (see Figure 10).

## 5 Conclusions

SARConnect does what it says on the box by providing AZ scientists with an interface that connect targets, activity and compounds from the major internal and external sources. The application exploits the web services of Chemistry Connect. Because it incorporates all human proteins, mapping gaps are restricted to residual curatorial ambiguity between Swiss-Prot IDs and a small proportion of target assignments made by the sources. While the target classification we developed is a critical component, we do not present our solution as necessarily ‘correct’. Nonetheless, we hope that the availability of analogous solutions in the public domain, together with resources such as the ConceptWiki that can align and maintain different target classification systems, will support expanding interoperability between both public and proprietary data sources.

The application is inherently flexible in that new target selections or chemical structure relationships can be added to the query interface. Crucially, it constitutes a de facto join between cheminformatics and bioinformatics. This means that scientists in our drug design teams can now execute advanced queries of the form “give me compounds for all the proteins in human pathway X associated with disease Y”. This reduces to a simple Swiss-Prot ID list with which the user can profile in SARConnect for active compounds and quickly select exact matches or close analogues from the AZ compound collection and/or chemical supplier catalogues. Subsequent mechanism of action (MOA) and potency analysis can rapidly progress target identification and validation.

The application has additional utilities beyond classical primary target-directed SAR. The first of these is a consequence of hypothesis-neutral and broad data capture providing a compound-protein interaction network where each pAct-to-protein link constitutes an edge. Chemical structures can thus be compared with those in the data set to provide useful information or inferences (e.g. cross-screening profiles, P450 and albumin binding), highlight potential assets (e.g. polypharmacology, repurposing, drug combinations or target-hopping) as well as indicate liabilities (e.g. hERG and other safety or side-effect related proteins). This network also facilitates the selection of chemical biology probes that can be used for perturbing normal and/or disease related pathways in any model system. This allows mechanistic hypotheses to be tested before progressing to target validation.

New targets under consideration by AZ or academic collaborators can be assessed for any type of relationship (homology-based or mechanistic) with proteins already connected to active compounds. The continually expanding range of chemotypes that Chemistry Connect feeds into SARConnect consequently provides a de facto chemical tractability assessment.^48^ It may even be adequate to iteratively generate results sufficient for disease model testing without the necessity to schedule an HTS. This principle can be extrapolated in two dimensions. The first is that as the range of targets with data occupancy expands, the probability of finding starting points for any new target (or a new MOA for an existing target) increases. The second dimension is that this probability of screening success increases still further as new bioactive scaffolds appearing from external sources are added to the compound collection.
