# Supplementary material for: SARConnect: A Tool to Interrogate the Connectivity Between Proteins, Chemical Structures and Activity Data
Source: Mol Inform. 2012 Aug 7;31(8):555–68. doi: 10.1002/minf.201200030 (PMC3535785; doi:10.1002/minf.201200030)
Supplement: Supplementary file 1 [file minf0031-0555-SD1.pdf]

SARConnect: A tool to interrogate the connectivity between proteins, chemical structures and activity data

Mats Eriksson, Ingemar Nilsson, Thierry Kogej, Christopher Southan, Martin Johansson, Christian Tyrchan, Sorel Muresan, Niklas Blomberg and Marcus Bjäreland

Supporting Information

Sources for the protein hierarchy system

Enzymes

The Enzymes are first gathered from EntrezGene by identifying all genes associated with an EC-identifier. The entries are then enriched with hierarchical data collected from the International Union of Biochemistry and Molecular Biology (IUBMB) database.

Even though EC-identifiers may contain up to four levels of information, we discard the fourth level as it denotes the actual leaf in the hierarchy tree and hence is not a class.

The protein family data is gathered from UniProt.

GPCRs

To collect the GPCR hierarchies two sources are used. The International Union of Basic and Clinical Pharmacology (IUPHAR) Committee on Receptor Nomenclature and Drug Classification database was deemed as the richest available source of GPCR hierarchies and was selected as the primary source. However several expected GPCRs was not covered at the time so GPCR DB was also selected as a complementing source.

The protein family data is gathered from UniProt.

Ion Channels

The International Union of Basic and Clinical Pharmacology (IUPHAR) Committee on Receptor Nomenclature and Drug Classification database contained all expected Ion Channels and were selected as the single source for the hierarchies.

The protein family data is gathered from UniProt.

NHRs

For the NHRs no complete and exhausting source could be identified at the time and considering the low number of genes belonging to this class the entries and their hierarchies were instead manually compiled and verified to be classified correctly.

The protein family data is gathered from UniProt.

Other

No hierarchical data is collected for entries belonging to this class other than the protein family data coming from UniProt.

The Druggability Keyword System

The druggability related keywords are used independently of the hierarchy system. They may also overlap so that two or more may be assigned to the same target. For entries were no match can be found, the annotation 'Other' is used. All Keyword–Target associations are gathered from UniProt. The current version has the following keywords:

Binding, Kinase, Lipase, Phosphatase, Protease, Signal, Transmembrane, Transport

Target database data points and their categorization

The data points are divided into the following categories.

1. Open Source Target data
2. Open Source Target Relation data
3. Company Internal Target data
4. Target Druggability data
5. Meta data
6. Administrative data

Open Source Target data

EGID (EntrezGene ID),

HGNCID (HUGO Gene Nomenclature Committee ID),

EGSYM (EntrezGene Official Symbol),

HGNCSYM (HUGO Gene Nomenclature Committee Official Symbol),

NAME (EntrezGene Long Gene Name),

PROTEIN_CLASS (Major Target Class),

TOP_FAMILY (UniProt Keyword Classes),

LEVEL1 (Hierarchical target level 1),

LEVEL2 (Hierarchical target level 2),

LEVEL3 (Hierarchical target level 3),

ALL_ECNUMBERS (EC-numbers from all sources combined),

EG_ECNUMBER (EC-numbers from EntrezGene),

UNIPROT_ECNUMBER (EC-numbers from UniProt),

BRENDA_ECNUMBER (EC-numbers from Brenda),

FIRST_UNIPROT_ACC (Accession ID from UniProt),

UNIPROT_FAMILY (UniProt Protein Family Name),

UNIPROT_ID (UniProt ID)

Open Source Target Relation data

INTERPRO_IDS (InterPro IDs linked to the target),
GENEONTOLOGY_IDS (Gene Ontology IDs linked to the target),
PDB_IDS (Protein Data Bank IDs linked to the target)

Company Internal Target data

UGENEID (Company Internal Gene Vocabulary ID),
GCID (Company Internal Gene Catalogue ID),
IBIS_SUB_TYPE (Company Internal Target Class Annotation),

Target Drugability data (Description is for positive annotation)

IS_A_DRUG_TARGET (Is targeted by approved drugs according to a set union of the Overington,TTD,GVKBio sources) ,
IS_A_PROJECT_TARGET (Is or have been a target in a company internal project),
IS_A_DRUGBANK_TARGET (Is annotated as a drug target in Drugbank),
PROJECT_TARGET_STATUS (Company Internal project status),
HAS_EGID_IN_IBIS (Has been tested against a compound company internal),
HAS_EGID_IN_GOSTAR (Has an EntrezGene entry in GVK BIO Online Structure Activity Relationship Database)

Meta Data (Description is for positive annotation)

HAS_SYMBOL_AMBIGUITY (The EntrezGene and HGNC official symbols are not identical),
MULTIPLE_CLASSES (Is a member of more than one major target class),
MULTIPLE_TOP_FAMILIES (Is a member of more than one UniProt Keyword Class),
MULTIPLE_GCID (Has multiple IDs in the Company Internal Gene Catalogue)

Administrative Data

RUNDATE (Date for latest data update)
